# The heterogeneity of plasma miRNA profiles in hepatocellular carcinoma patients and the exploration of diagnostic circulating miRNAs for hepatocellular carcinoma

**DOI:** 10.1371/journal.pone.0211581

**Published:** 2019-02-05

**Authors:** Xue Bai, Zhenzhen Liu, Xiaojian Shao, Di Wang, Encheng Dong, Yan Wang, Chung-I Wu, Yunfei Yuan, Xuemei Lu, Chunyan Li

**Affiliations:** 1 Key Laboratory of Genomic and Precision Medicine, Beijing Institute of Genomics, Chinese Academy of Sciences, Beijing, China; 3 Key Laboratory of Oncology in South China/Department of Hepatobiliary Oncology, Sun Yat-sen University Cancer Center, Guangzhou, Guangdong, China; 2 Beijing Anzhen Hospital, Capital Medical University, Beijing, China; 4 Center for Excellence in Animal Evolution and Genetics, Chinese Academy of Sciences, Kunming, Yunnan, China; Mayo Clinic in Arizona, UNITED STATES

## Abstract

Heterogeneity is prevalent in cancer both between and within individuals. Although a few studies have identified several circulating microRNAs (miRNAs) for cancer diagnosis, the complete plasma miRNA profile for hepatocellular carcinoma (HCC) remains undefined, and whether the plasma miRNA profiles are heterogeneous is unknown. Here, we obtained individualized plasma miRNA profiles of both healthy subjects and HCC patients via genome-wide deep sequencing. Compared with the highly stable miRNA profile of the healthy subjects, the profile of the HCC patients was highly variable. Seven miRNAs were optimized as potential plasma-based biomarkers for HCC diagnosis. Combined with the clinical data of The Cancer Genome Atlas (TCGA) cohort, three out of the seven miRNAs were correlated with the survival of the HCC patients. To investigate the effect of cancer cells on the plasma miRNAs profile, we compared the most differentially expressed miRNAs between plasma and tissues. Furthermore, miRNAseq data of HCC patients from TCGA were recruited for comparisons. We found that the differences between plasma and tissue were inconsistent, suggesting that other cells in addition to cancer cells also contribute to plasma miRNAs. Using two HCC cancer cell lines, we examined the levels of seven differentially expressed miRNAs. The reverse direction of certain miRNAs alterations between cancer cells and media further confirmed that miRNAs may be selectively pump out by cancer cells.

## Introduction

Cancer heterogeneity has been recognized as an important clinical determinant of patient outcomes, such as response or resistance to anti-cancer therapies [1,2]. High-resolution genome-wide studies have revealed that heterogeneity is prevalent in cancer, not only in different individuals, but also within the same tumor [3–6]. Biomarkers are required to guide clinical decisions for cancer therapy based on the inter- and intratumor heterogeneity. For the clinical diagnosis using tissue, one of the obvious challenges is the representation bias of the biopsies [4]. Since plasma is more accessible and easier to process and microRNAs (miRNAs) are highly stable in blood, plasma miRNA profile analysis will indisputably open a new era in biomarker research and provide a new paradigm for non-invasive characterization of cancer, both for diagnosis and therapy monitoring.

Lu et al. successfully classified poorly differentiated tumors using miRNA expression profiles [7]. Their findings demonstrated that miRNAs can be used as biomarkers for cancer diagnosis. miRNA is highly prevalent not only in tissues but also in body fluids, including plasma, serum, sputum, and urine. miRNAs are stable in blood and can be reliably detected [8–10]. Mitchell et al. found that tumor-derived miRNAs were detectable in plasma from a mouse prostate cancer xenograft model and in clinical serum specimens from prostate cancer patients [10]. Chen et al. developed the next-generation sequencing to characterize miRNAs in serum for diagnosis of lung cancer and diabetes [8]. However, in their study, the serum samples of each group were pooled for further analysis. Cancer heterogeneity has been well studied at the DNA, RNA, protein, and even microenvironment levels [11,12]. But it is unclear whether there is heterogeneity in serum/plasma miRNA profiles between cancer patients. Sample pooling of serum/plasma may lead to false-positive or false-negative results because of the inter-individual variations. With the development of sequencing technology, it is feasible to obtain individual plasma miRNA profiles. Since dysregulation of miRNAs is considered an early event in tumorigenesis, miRNAs are promising biomarkers for early diagnosis of cancer [13,14].

Liver cancer is the fifth most common cancer worldwide, but it was the second most frequent cause of cancer-related death worldwide for men in 2013 [15]. Hepatocellular carcinoma (HCC) is the major type of primary liver cancer. Additionally, 55% of all HCC cases worldwide occur in China every year, and the mortality is high [16,17]. However, most HCC cases are diagnosed at the advanced stage, leading to the poor survival rate in China [18]. In addition, the recurrence rate is up to 50% within two years [19,20]. Late diagnosis of HCC at the advanced stage leads to a poor therapy response; thus, a more efficient diagnosis for early HCC is demanded [21]. Alpha fetoprotein (AFP) detection in the serum and liver ultrasonography (US) are the routinely used tools for HCC diagnosis. The sensitivity and specificity of AFP for HCC are 39%-65% and 76%-94%, respectively, and the positive prediction value of AFP varies from 9% to 50% depending on the cut-off value [22]. In addition, multiple factors, such as gender and viral etiology, should be considered when using the serum AFP level for HCC diagnosis or prognosis [23]. A combination of AFP and DCP (des-γ-carboxyprothrombin) improves the sensitivity of HCC diagnosis to 84%, which still requires improvement [18]. The performance of US varies depending on the manipulators. Generally, for US, the sensitivity is higher than 60% and the specificity is greater than 90%, but the positive predictive value is 70% [22]. Above all, it is urgent to explore more reliable biomarkers for HCC diagnosis and surveillance.

Little is known about the origin of the extracellular circulating RNA [13,24]. Mitchell reported that serum-derived miRNAs originated from prostate cancer tissues [10]. Although miRNAs are released from cells in a cell type-dependent fashion, the miRNA repertoire may differ from that of the origin cells [25,26]. Whether circulating miRNAs are actively secreted by cells or are occasionally derived from lysed cells by a passive release mechanism is uncertain [8,14].

To exclude the individual variability, plasma miRNA was obtained and sequenced individually in this study. Here, we present the plasma miRNA expression profiles of 10 male HCC patients and 4 healthy subjects obtained via Illumina massively parallel sequencing. We attempted to clarify the modulation of miRNAs release by comparing the enrichment or elimination of miRNAs in tissue and plasma or in cells and media.

## Materials and methods

### Patients, plasma preparation, and tissue preparation

A study population of ten HCC patients was enrolled at the Sun Yat-sen University Cancer Center (Guangzhou, China). The diagnosis of HCC was confirmed by histology. Pre-surgical blood samples from the HCC patients and healthy subjects were collected in EDTA-vacuum tubes (BD, Polymouth, UK), and plasma was isolated within 30 min. The blood collection tubes were centrifuged at 500 g for 10 min at 4°C, the supernatant layer was transferred to new RNase-free tubes, and the samples were further centrifuged at 1800 g for 10 min at 4°C to prepare plasma without cell debris. Corresponding pairs of primary HCC tissues and the adjacent non-tumor tissues were obtained from the patients undergoing resection at Sun Yat-sen University Cancer Center. The tissues were immediately treated with RNA-later (Ambion, Austin, TX), and stored at -80°C before use. All study subjects provided written consent, and the present study was approved with the statement (2012H007) by the Human Research Ethics Review Board of Beijing Institute of Genomics, CAS.

### Plasma small RNA sequencing

Total RNA was isolated from plasma using TRIzol reagent (Invitrogen, Carlsbad, CA). Small RNA was size fractionated by polyacrylamide gel electrophoresis, and a small RNA fraction ranging from 18 to 30 nt was excised. RNA eluted from the polyacrylamide gel was prepared for library construction according to the Illumina Small RNA Sample Prep protocol. The small RNA library was sequenced using HiSeq2000 (Illumina Inc., CA) to generate more than 6 GB of data per small RNA library. The sequencer images were processed to derive base calls and generate digital data. After removal of adaptor sequences, the reads were then used for bioinformatics analysis. The plasma miRNA sequencing dataset is available at Genome Sequence Archive (GSA) in BIG Data Center (http://bigd.big.ac.cn/) under the accession number CRA000807 (or BioProject PRJCA000794).

### Plasma miRNA sequencing data analysis

Perl scripts were developed to change the FASTQ data to FASTA files. Additionally, the sequences of the 3 ′ adaptor with barcode information were cleaned up. The remaining sequences were filtered, and sequences with lengths ≥ 18 nt were mapped using BLASTN against both the human miRNA sequences (downloaded from miRBase, version 21) and human transcript reference sequences (downloaded from the NCBI ftp site). Because the lengths of miRNAs are similar, the reads counts of each miRNA was normalized by the total number of miRNA reads generated in the library for each subject, and then, the ratio was multiplied by a constant (1 × 10^6^). The reads per million (RPM) values of each miRNA for each individual are listed in S1 Table. The miRNAs with an RPM of more than 10 were considered to be expressed [27]. To compare the differential expression of miRNAs in plasma between the HCC patients and healthy subjects, R package DESeq was used with a cut-off of fold change > 2 and *P*adj < 0.05.

### Comparison of Pearson’s correlation coefficient (R)

Pearson’s correlation coefficient (R) is used to measure the strength of the association between two variables. An ANOVA (analysis of variance) *F*-test was performed to calculate the difference between these two groups of the HCC patients and the healthy subjects.

### The Cancer Genome Atlas (TCGA) tissue miRNA expression analysis between tumor (T) and adjacent normal tissue (N) from HCC patients

The miRNA expression data of the cancer tissues from HCC patients were downloaded from TCGA (https://portal.gdc.cancer.gov). To compare the expression levels of miRNAs from the plasma we sequenced, the miRNA expression data from TCGA were re-analysed. First, the mapping reads were recounted according to the miRNA sites from miRBase (version 21). The start position matched perfectly, while the 3' mapping region ranged between -1 and +3. Next, reads mapping to the same miRNAs were pooled. R package “DESeq” was used to characterize the differentially expressed miRNAs between T and N with a cut-off fold change > 2 and *P*adj < 0.05 [28]. A clustered heatmap was generated with R package “pheatmap” based on the normalized expression level, and Pearson’s correlation method was used to calculate distances.

### The association study of seven potential biomarkers with patients’ survival

miRNA expression data and clinical data of HCC patients were downloaded from TCGA. For the seven miRNAs, we analyzed miRNA with the RPM more than 10 in at least 10% of the samples in TCGA cohort. The samples were divided into two groups according to the upper and lower quartile expression of the miRNA. Only the miRNAs with more 5 samples in each group were used for further analysis. The R package ‘survival’ is used to calculate and plot Kaplan–Meier survival curves. *P* values for survival curves were determined by use of the log-rank test.

### Fold change comparison of the differentially expressed genes between tissue and plasma

The fold change values between tissues were obtained by pairwise comparisons of tumor and normal tissues. For plasma, the values were calculated for each HCC sample compared with the average of the healthy samples. If the denominator was equal to 0, we added 0.01. The box diagrams illustrated the fold changes of the differentially expressed miRNAs in plasma or tissue, including miRNAs with an RPM more than 10 in at least 10% of the samples. The diagrams were ordered by the median value of the fold change values. Boxplot edges indicate the 25^th^ and 75^th^ percentiles, and whiskers indicate nonoutlier extremes.

### RNA extraction from tissues, cells, and HCC cell medium

Total RNA was isolated using TRIzol reagent (Invitrogen, Carlsbad, CA) according to the manufacturer's instructions. Two HCC cancer cell lines (HepG2 and Huh7) were used in this study, and the hepatocyte cell line L02 was used as control. Next, 5 × 10^6^ cells were plated in a 10 cm dish with 5 ml of medium (HepG2: MEM; Huh7: DMEM; L02: DMEM). Twenty-four hours later, the cells and medium were collected separately. The medium was centrifuged at 500 g for 10 min, and then, the supernatant was transferred into new RNase-free tubes and centrifuged at 2,000 g for another 10 min. The supernatant was used for total RNA extraction. For each cell line, ~4 ml of medium was collected. The concentration was determined using a Nanodrop 2000 spectrophotometer (Thermo, Wilmington, DE), and the integrity of the RNA was examined by gel electrophoresis. Next, 500 ng total RNA for each sample was reverse transcribed using a miScript II RT Kit (Qiagen, Hilden, Germany), and 20 μl of the obtained cDNA was diluted into a total volume of 100 μl.

### Quantitative reverse transcription-polymerase chain reaction (qRT-PCR)

For each qRT-PCR experiment, 1 μl of diluted cDNA was used as the template. Quantification of mature miRNAs was performed using Maxima SYBR Green/ROX qPCR Master Mix (Thermo, Eugene, OR) in triplicate 20 μl reactions according to the manufacturer’s protocol with an Applied Biosystems 7500 real-time PCR system. Thermal cycling was organized into 2 steps: a first denaturation step of 10 min at 95°C, followed by 40 repeated cycles of 95°C for 10 sec and 60°C for 31 sec. U6 snRNA was used as the endogenous control for tissues, cells, and the medium. qRT-PCR forward primers for miRNA were ordered from Guangzhou RiboBio Co., Ltd. (Guangzhou, China). The qRT-PCR reverse and forward primers for U6 snRNA were from a miScript SYBR Green PCR Kit (Qiagen, Hilden, Germany). The expression level of each miRNA was normalized to that of the control. The ΔΔCt method was used to measure the expression level of each miRNA. ΔCt = Ct (target)− Ct (control). ΔΔCt = ΔCt (tumor)– ΔCt (non-tumor) or ΔΔCt = ΔCt (HCC cell line)– ΔCt (L02). The fold change of each miRNA was calculated using the 2^−ΔΔCt^ method.

## Results

### The plasma miRNA expression profile is more variable among HCC patients than that among healthy subjects

**Fig 1:**
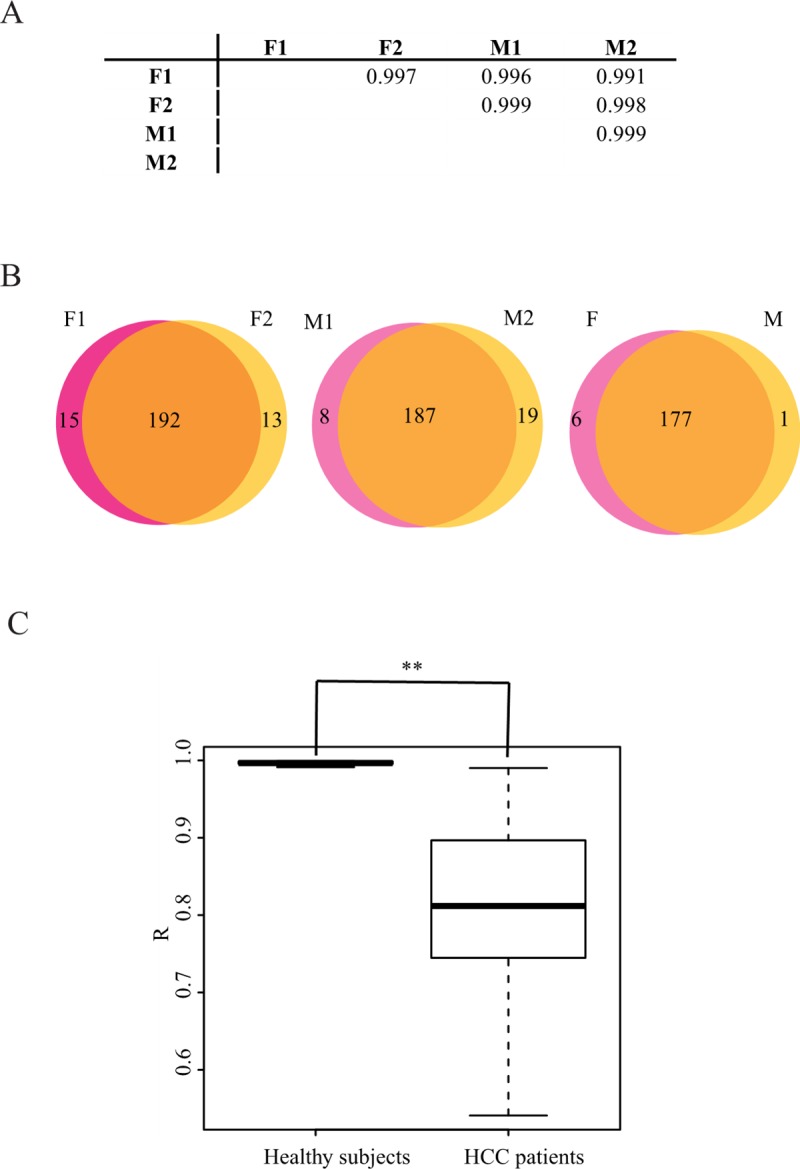
The plasma miRNA profiles are much more variable for HCC patients than those for heathy subjects. A. The correlation coefficients for the pairwise comparison of healthy subjects are close to 1.0 between individuals. F indicates female, and M indicates male. B. The plasma miRNAs have minor gender bias among healthy subjects. miRNAs with an RPM more than 10 were used to analyse the types of miRNAs in the plasma. The types of differential miRNAs between genders are comparable to those within gender. C. Comparison of the Pearson’s correlation coefficient (R) comparison between healthy subjects and HCC patients. The differentiation between these two populations is significant. The *P* value is less than 0.001.

We adopted a genomic approach to survey signature of plasma miRNA profiles in an unbiased manner. This is the first study to sequence the plasma miRNAs individually, which is an efficient means to clarify the differences among subjects. First, the plasma miRNA profiles of healthy subjects were deciphered. In our dataset, two plasma samples were sequenced for each gender. R was close to 1.0, both between and within the gender groups (Fig 1A). The expression level of circulating miRNAs was quite stable among the healthy subjects, which is consistent with the observations of Chen et al. [8]. We further dissected the types of miRNAs detected in female or male plasma. The Venn diagram shows that the types of differentially expressed miRNAs between genders were comparable to the types within genders (Fig 1B). Therefore, plasma miRNAs exhibited a minor gender bias among healthy subjects. Taken together, the results showed that the plasma miRNA expression profile is stable among healthy subjects.

Accumulated deep-sequencing data have demonstrated that there is heterogeneity in cancer, both between and within subjects [29,30]. Is there heterogeneity in the plasma miRNA expression profiles of cancer patients? To minimize gender bias, small RNA libraries from 10 male HCC patients were constructed individually and were sequenced with barcodes in this study. The distribution of R (correlation coefficient) in the HCC patients had a broad range from 0.541 to 0.990, with a median of 0.812 (Fig 1C). This comparison revealed that the plasma miRNA expression profile of HCC patients is much more variable than that of healthy subjects.

### Potential biological function of the plasma miRNAs

**Fig 2:**
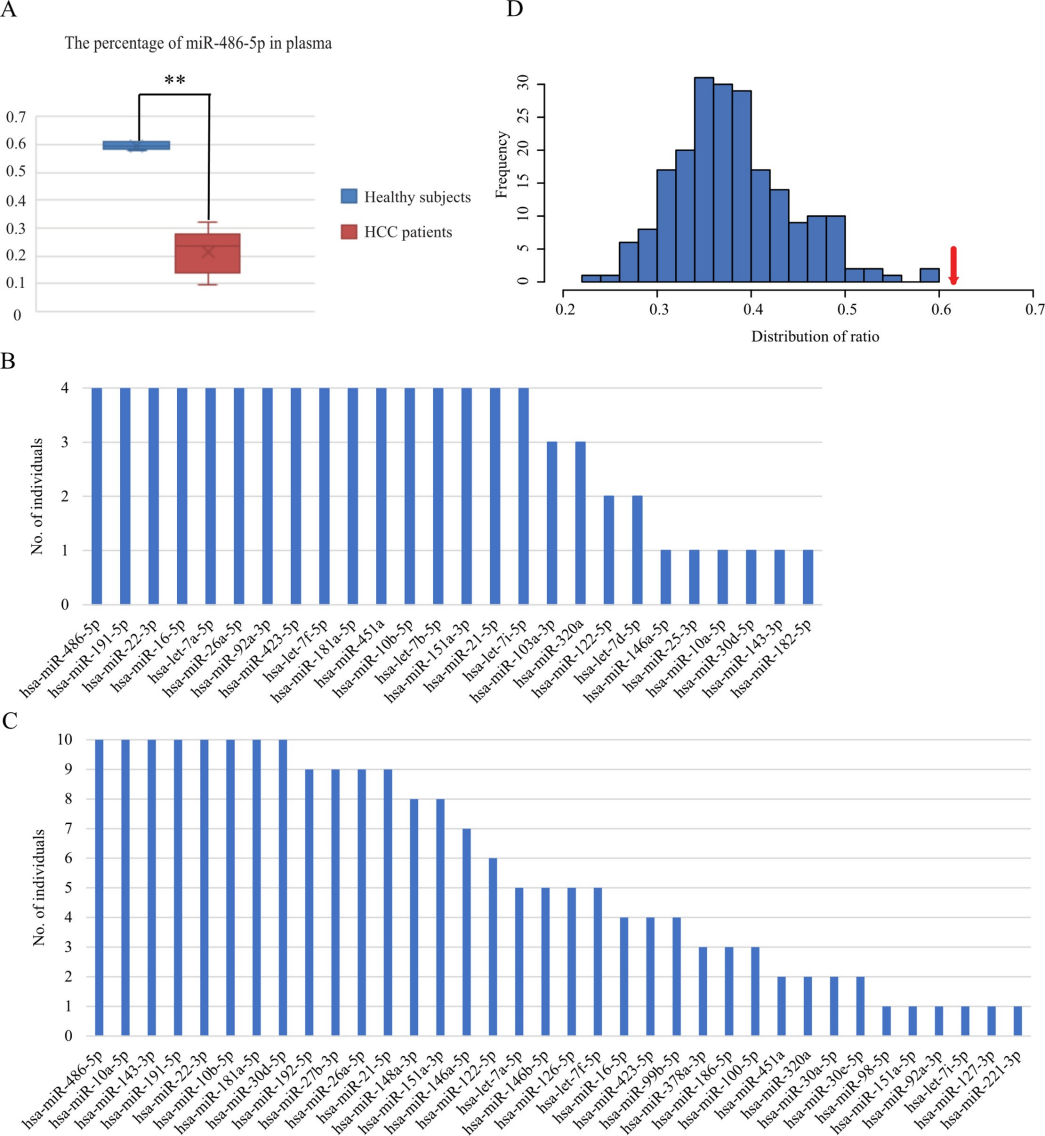
Most abundant miRNAs in plasma. A. miR-486-5p is more abundant in the plasma of healthy subjects. B. The collected dataset of the top twenty most abundant plasma miRNAs in healthy subjects. C. Collected dataset of the top twenty most abundant plasma miRNAs in HCC patients. D. Distribution of the ratio of the miRNAs shared by all the individuals. Four individuals among ten HCC patients were randomly sampled for two hundred and ten times. The red arrow indicates the ratio of the healthy subjects.

The biological function of the circulating miRNAs in healthy subjects is entirely unknown. Both miRNAs with predominant oncogenic properties (such as miR-21-5p) and miRNAs with tumor suppressor properties (such as the let-7 family) can be found in the plasma of healthy subjects. The most abundant miRNAs may direct the biological function of the miRNAs in plasma. Most strikingly, the reads of miR-486-5p account for ~60% of the total reads mapping to miRNAs in healthy subjects. Interestingly, the abundance of miR-486-5p was dramatically lower (*P* < 0.001) in the plasma of HCC patients (Fig 2A). Three independent studies also found miR-486-5p to be relatively abundant in the blood of healthy subjects [31–33]. One hundred and ten genes are potential targets of miR-486-5p, as predicted by miRanda, miRDB, miRwalk, and TargetScan (S2 Table). Gene-annotation enrichment analysis of the targets suggested that miR-486-5p is mainly involved in the regulation of cell proliferation (S3 Table).

To investigate the potential biological function of the plasma miRNAs, we screened out the top twenty abundant plasma miRNAs for each individual. For the healthy subjects, 16 of the 26 abundant miRNAs were shared between all of the 4 subjects (Fig 2B and S4 Table). Through Ingenuity Pathway Analysis (IPA), 392 genes were experimentally validated as the targets of these 16 miRNAs (S5 Table). Gene-annotation enrichment analysis of these genes suggested that the most abundant miRNAs regulate basic cellular processes, such as metabolic processes, cell death, and apoptosis (S6 Table). However, for the HCC patients, only 8 of the 36 abundant miRNAs were shared by all the 10 samples (Fig 2C and S7 Table). All the eight miRNAs were also shared by all the healthy subjects. Target prediction for these eight miRNAs and functional analysis of the targets were performed via IPA. In addition to transcription regulation, the targets were mainly involved in the cellular metabolic processes (S8 Table), which are critical to cancer development.

To eliminate the effect of sample size between healthy subjects and HCC patients, four individuals were randomly sampled from the 10 HCC patients for two hundred and ten times based on complete enumeration of all possible combinations. The percentage of miRNAs shared by the 4 individuals is presented in Fig 2D. The healthy subjects had the highest percentage (61.5%, denoted by the red arrow) among the comparisons, further confirming that the plasma miRNA profiles were more divergent among the HCC patients.

### Differentially expressed plasma miRNAs between the HCC patients and the healthy subjects are potential biomarkers for HCC diagnosis

**Fig 3:**
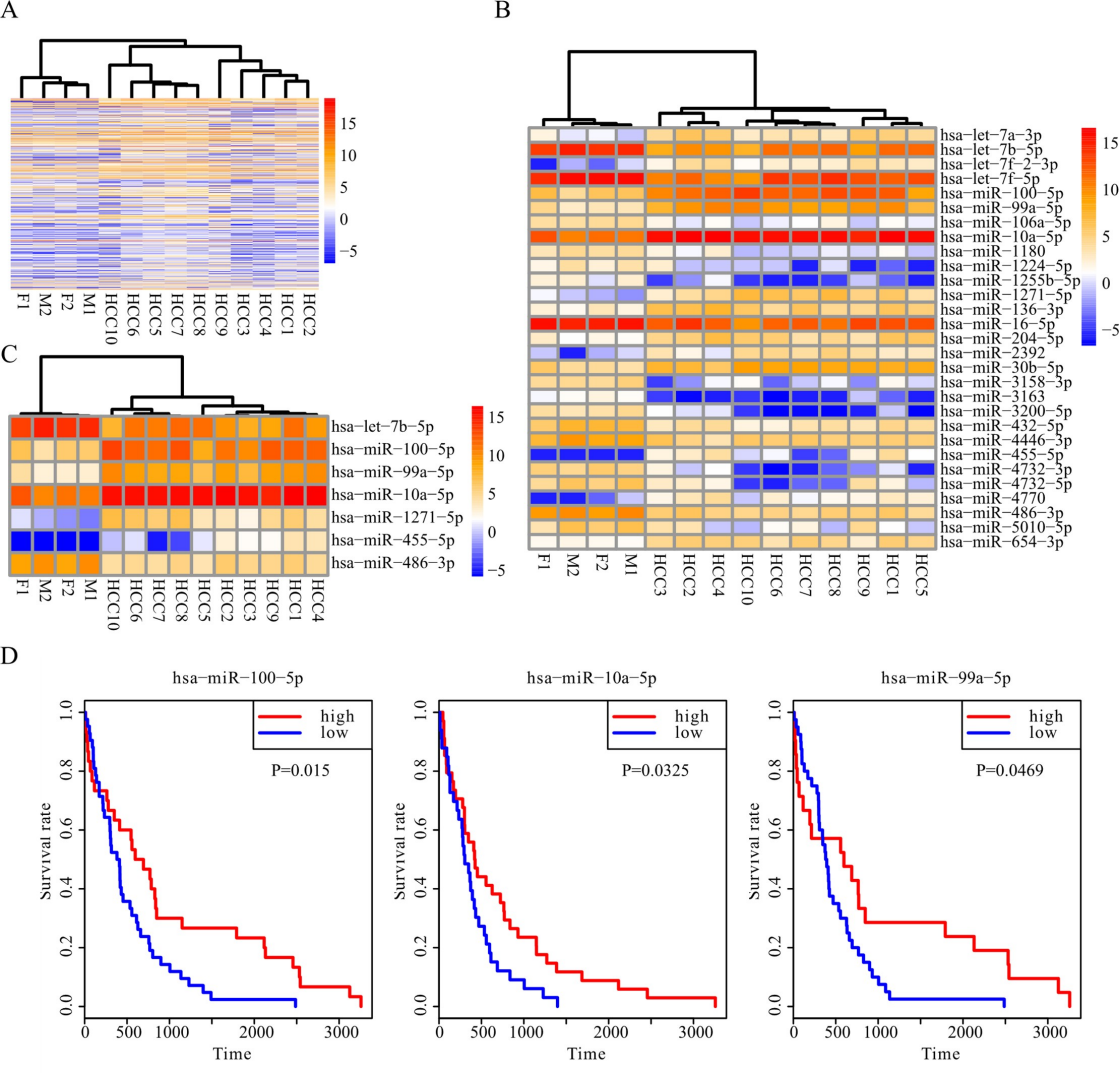
Differentially expressed miRNA in plasma between HCC patients and healthy subjects. A. The plasma miRNA profiles of HCC patients and healthy subjects are different. B. The twenty-nine most differentially expressed miRNAs can differentiate HCC patients from healthy subjects. C. The seven miRNAs predicted to be the biomarkers for HCC diagnosis can differentiate HCC patients from healthy subjects. D. Kaplan-Meier plots for patient stratification based on the expression of three of the seven miRNAs (the same as C). The gene names of each miRNA are annotated above each plot.

We compared the expression profiles of plasma miRNAs between the HCC patients and the healthy subjects and found that the individuals were clustered into two groups (Fig 3A). The differential profiles between these two groups confirmed that the plasma miRNAs are potential diagnostic biomarkers for HCC. The statistics of the fold change for each miRNA are shown in S9 Table. The most increased or decreased miRNAs in the plasma of HCC patient plasma compared with those in the healthy subjects are more likely to be diagnostic biomarkers for HCC. The miRNAs with log_2_ (fold change) > 1 or < -1, and *P*adj < 0.05 were recruited as potential diagnostic HCC biomarkers. Using the cluster of these 29 miRNAs, HCC patients could be distinguished from the healthy subjects (Fig 3B). Regarding the application of plasma miRNAs as HCC diagnosis biomarkers, if the expression level is too low in the plasma, the accuracy of these biomarkers will be low using canonical measures for miRNA quantitation, such as qRT-PCR. We screened twenty-one miRNAs with an expression level higher than 10 (baseMean_HCC patient by DESeq in S9 Table) in HCC patients, and then chosen seven of the most variant miRNAs (5 up-regulated and 2 down-regulated in HCC patient plasma) as the potential diagnostic biomarkers for HCC. A panel of these seven miRNAs was sufficient to distinguish the HCC patients from the healthy subjects (Fig 3C). A larger panel of studies is required to validate whether these seven plasma miRNAs are optimal diagnostic biomarkers for HCC in the future. To validate that the seven miRNAs could be used as the biomarkers for HCC, the miRNASeq data of tissue and the clinical data of TCGA HCC cohort was re-analyzed. Three out of the seven miRNAs are correlated with the survival of HCC patients in the TCGA cohort (Fig 3D), which further confirms that the circulating miRNAs in plasma may be used as diagnostic biomarkers for HCC.

### Cancer cells may selectively release miRNAs into the plasma

**Fig 4:**
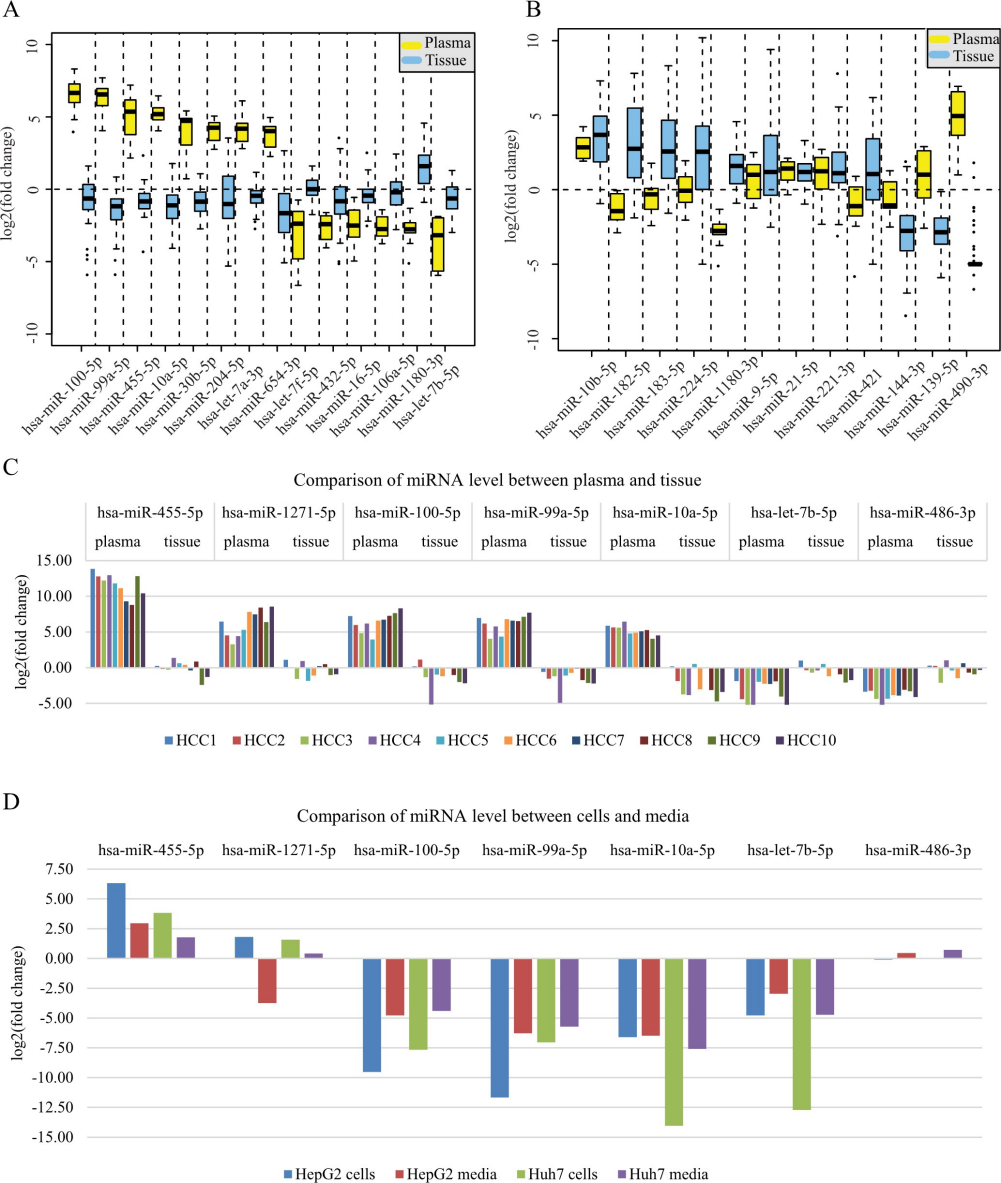
Comparisons of miRNAs between plasma and tissue and between cells and media. A. Fold change of miRNAs that are most differentiated in plasma (in yellow) and that can be detected in tissues of TCGA dataset (in blue). B. Fold change of miRNAs that are most differentiated in tissues of TCGA dataset and that can be detected in plasma. C. Expression of the seven biomarkers in plasma and tissue within the same individuals. D. Expression of the seven biomarkers in the HCC cell lines (HepG2 and Huh7) and media.

If the cancer cell releases all its miRNA into the plasma without selection, it is more likely that the miRNAs enriched in cancer cells will be up-regulated in the plasma. To examine the effect of cancer cells on the plasma miRNA profile, we screened out the twenty-nine most differentially expressed miRNAs in plasma between the HCC patients and the healthy subjects, and then analyzed their expression level in tissue using paired tumor and normal tissue miRNASeq data from HCC patients in TCGA, as described in the Materials and Methods. Among these twenty-nine miRNAs, fourteen miRNAs were detected in the TCGA dataset. Interestingly, eight of the fourteen miRNAs were up-regulated in the plasma of HCC patients, while all of the eight miRNAs were down-regulated in the tumors (Fig 4A). Additionally, miR-1180-3p, which was up-regulated in the tumors, was down-regulated in the plasma of HCC patients (Fig 4A). We further selected the most differentially expressed miRNAs between T and N in TCGA dataset and screened out twelve miRNAs that were detected in plasma using the criteria described in the Materials and Methods. Seven of the twelve miRNAs showed reverse changes between plasma and tissue (Fig 4B). The contrary trends between the plasma and tissue indicate that cancer cells may selectively release miRNAs into the plasma, although the mechanism is unknown.

To compare differences in the plasma and tissue miRNAs from the same individual, we quantified the expression level of the seven miRNAs predicted to be biomarkers for HCC diagnosis in the T and N tissues of the ten HCC patients whose plasma miRNAs were sequenced in this study. Contrary trends still existed within individuals, including miR-100-5p, miR-99a-5p, and miR-10a-5p levels (Fig 4C).

In addition to cancer cells, other cells, such as the circulating blood cells, also secrete miRNAs into the plasma [8]. To eliminate the interference from other cells, cancer cell lines were used to determine the effect of cancer cells on the extracellular miRNA profiles. If miRNAs are not selectively released by cancer cells, the alterations in miRNAs between cells and the medium should be positively correlated. In this study, we used the hepatocyte cell line L02 as control cells and the HCC cell lines HepG2 and Huh7 to investigate differences in miRNA expression between the medium and cells. The expression changes of the seven miRNAs, which are supposed to be diagnostic biomarkers for HCC, were pairwise compared between the medium and the cells for HepG2 and Huh7 cells. For the five miRNAs in both the HCC cell lines, the directions of the miRNA alterations were consistent between the medium and the cells; however, for miR-1271-5p, the alteration direction was reversed in HepG2 cells, and for miR-486-3p, the alteration direction was slightly reversed in both HepG2 and Huh7 cells (Fig 4D). Furthermore, we examined another four miRNAs and found more miRNAs have reverse change between cells and medium (S10 Table). Thus, cancer cells may selectively secrete miRNAs into the medium.

## Discussion

Cancer is a heterogeneous disease, both between individuals and within the tumor, but it is unclear whether there is heterogeneity in plasma miRNA profiles between cancer patients. This study examined the individual miRNA repertoires of healthy subjects and HCC patients. We found that the miRNAs profiles of HCC patients were much more variable than those of healthy subjects. A plasma or serum pooling strategy may increase the false-positive or false-negative ratio during initial biomarker exploration. To the best of our knowledge, this is the first study to analyse the plasma miRNA profiles individually, without a blood mixture.

Circulating miRNAs have been explored as non-invasive cancer diagnostic or prognostic biomarkers for lung, colorectal, and prostate cancer [8,10,30]. Although several studies have attempted to characterize potential diagnostic biomarkers via qRT-PCR validation of several candidate miRNAs in HCC cohorts, no circulating miRNAs have been characterized and validated as a standard reference for HCC [34–37]. In this study, we attempted to provide biomarker candidates for HCC without bias using genome-wide screening. Because the sample size was small in this study, larger scale validation studies are required to develop miRNA combinations to improve the specificity and sensitivity for HCC diagnosis.

Why miRNAs are present in plasma is still unknown. Thus far, plasma miRNAs are generally regarded as a mixture of miRNAs released from cells of different tissues in the body and estimating how many miRNAs are released from the cancer cells is difficult. The miRNA expression level was compared between the plasma and the tissue of the same individuals, which may provide hints to the sources of the miRNAs in plasma. Furthermore, we used HCC cancer cell lines (HepG2 and Huh7) to avoid the interference from the other cells in the body, and we found that cancer cells may selectively to release miRNAs. Additionally, other cells other than cancer cells must also secrete miRNAs into plasma.

Previous investigations have suggested that miRNAs transferred in exosomes may mediate intercellular communication [26,38]. Functional analysis of these miRNAs in cancer cells will lead to insights into cancer biology. Of the seven potential HCC biomarkers, the expression level of miR-486-3p was quite low in the tumor tissue based on TCGA miRNASeq data. Many studies have reported that the remaining six miRNAs function as tumor suppressors to inhibit cell proliferation, migration and invasion of cancer cells [39–47]. Cancer cells might pump these microRNAs into the circulatory system to eliminate their ability to negatively control tumor growth.

## Supporting information

S1 Table The expression of miR for all the plasma samples. (XLSX)

S2 Table The predicted targets of miR-486-5p. (XLSX)

S3 Table Gene-annotation enrichment analysis of the potential targets of miR-486-5p. (XLSX)

S4 Table The 20 most abundant miRNAs for each healthy subject. (XLSX)

S5 Table The predicted targets of the most abundant miRNAs in all the 4 healthy subjects. (XLSX)

S6 Table Gene-annotation enrichment for the targets of the abundant miRNAs in the plasma of healthy subjects. (XLSX)

S7 Table The 20 most abundant miRNAs for each HCC patient. (XLSX)

S8 Table Gene-annotation enrichment for the targets of the most abundant miRNAs in all the HCC patients. (XLSX)

S9 Table The statistical analysis of the fold change for each miRNA by DESeq. (XLSX)

S10 Table The fold change of another four miRNAs in cells and media. (XLSX)
